# Diseases of the Abdomen

**Published:** 1902-11-15

**Authors:** 


### Diseases of the Abdomen.

(*Continued from page* 97.)

Michael Foster,[8] in commenting upon the spurious intestinal sand, states that in his experience large clay-coloured stools are usually associated with the passage of such sand of vegetable origin. During the passage there is fever and some symptoms of intestinal obstruction. He further discusses the question of the association of appendicitis and membranous colitis. One would naturally consider that as the appendix is part of the colon mucous membrane the morbid process of the colon would tend to involve the appendix. Dieulafoy controverts this by reasoning of the rarity of appendicitis in enteric or tubercular ulceration. Foster has twice seen cases where this complication was met with, one where an operation was performed and the appendix found full of mucus. In the second there was a discharge of fœtid pus by the bowel, which preceded by three months the passage of membrane which continued for three years. These cases only serve to reveal the risk of setting on one side the possibility of appendicitis because of the presence of intestinal casts. There are two types of sufferers from membranous colitis. They are both neurasthenic: (1) The dyspeptic, moody, and melancholic type; and (2) the busy, cheerful, and enthusiastic type, but prone to insomnia and with poor appetite. Both are busy hard workers, using their energy at too rapid a rate. He considers from the study of cases that neurasthenic symptoms have been present before the colitis, and that it must be regarded as a hypersecretion of mucus taking place under the influence of unknown conditions of the nervous system, and the amorphous secretion is converted into membrane through the concomitant loss of tone in the bowel. Besides neurotic elements gout plays an ætiological part in some cases, as in the case of Dieulafoy's intestinal sand. In seven cases it has developed in the subjects of healed phthisis; in six of these arrest had been present for from one to five years; while in the seventh an unhealed bronchiectasis was present.

In treating this affection he speaks favourably of castor oil in drachm doses frequently repeated, or soap and water enemata or clysters of ℥ iv.–vj. of olive oil. Bael fruit lessens colicky pains and constipation. Bismuth is of very little good. Opium is good in acute attacks, but iron is rarely tolerated, and only should be given after a blood count shows the need of it. Strychnine should be used with caution. Silver nitrate in $\frac{1}{32}$ gr. doses is sometimes of great service. The baths at Plombières are good: the water contains silica and arsenic, and is mildly alkaline and at a temperature of 51° C. Douches of the same ($\frac{3}{4}$ to $1\frac{1}{2}$ litre) are recommended with alterations of position during the administration to allow of the fluid reaching every part of the colon, and the external spray upon the abdomen under the water. He speaks highly of colotomy as instanced by one case, but it should never be long delayed.

Mott[9] considers that there is an asylum dysentery which is distinct from dysentery *in* asylums—not bacterial or of extrinsic origin, but associated with nerve degenerations.

Washburn and Owen Richards[10] recognise three types of dysentery:—(1) The normal with febrile condition lasting a week, and becoming chronic unless ending fatally; (2) mild, non-febrile attack, soon well; (3) the pyrexia continuing for three or four weeks, with perhaps entire absence of blood and mucus, the bowels being loose or constipated—a typhoid form due to septic invasion. The mortality in Africa was low (1·86 per cent. in the Imperial Yeomanry at Pretoria). Hepatic abscesses were rare, and with streptococci and bacilli unlike the tropical form. No amœbæ were to be found in the abscess or the intestine. The cæcum and rectum were most affected, the walls being thickened, and hæmorrhage and ulcers being present, perforation being the most frequent cause of death.

Other observers have failed to find amœbæ, though there is some disagreement as to where the inflammation starts. Rolleston holds that it commences in the mucous layers, Mott and Bruce in the submucous layers.

Some rare complications of dysentery are given by Remlinger.[11] He has met with an acute parenchymatous nephritis, associated with dysenteric muscular pains in lower extremities; a marked general anasarca, without evidence of its being of cardiac or renal origin; no albuminuria, but possibly some inherent change in the kidney, as the case got well under the usual treatment of nephritis; arthritis of the right knee-joint with fluid, the whole right lower extremity becoming swollen; epididymitis without gonorrhœa in two cases; and abscess of the spleen, leading to general peritonitis and death from rupture.

[8] Edinburgh Med. Rev., February. [9] Lancet, Feb. 1. [10] Lancet, Feb. 1. [11] Amer. Jour. Med. Sci., March.

(*To be continued.*)

